# The Role of Vitamin D in Hematopoietic Stem Cell Transplantation: Implications for Graft-versus-Host Disease—A Narrative Review

**DOI:** 10.3390/nu16172976

**Published:** 2024-09-03

**Authors:** Stefano Mancin, Giovanni Cangelosi, Sofia Matteucci, Sara Morales Palomares, Mauro Parozzi, Elena Sandri, Marco Sguanci, Michela Piredda

**Affiliations:** 1 IRCCS Humanitas Research Hospital, Via Manzoni 56, 20089 Rozzano, Italy; stefano.mancin@humanitas.it (S.M.); sofia.matteucci@humanitas.it (S.M.); 2 Units of Diabetology, ASUR Marche, Via Augusto Murri 21, 63900 Fermo, Italy; giovanni.cangelosi@virgilio.it; 3 Department of Pharmacy, Health and Nutritional Sciences (DFSSN), University of Calabria, 87036 Rende, Italy; sara.morales@unical.it; 4 School of Nursing, ASST Santi Paolo e Carlo, “San Paolo” Campus, University of Milan, 20142 Milan, Italy; 5 Faculty of Medicine and Health Sciences, Catholic University of Valencia San Vicente Mártir, c/Quevedo, 2, 46001 Valencia, Spain; elena.sandri@ucv.es; 6 Research Unit of Nursing Science, Department of Medicine and Surgery, Campus Bio-Medico di Roma University, Via Alvaro del Portillo 21, 00128 Rome, Italy; marco.sguanci@unicampus.it

**Keywords:** vitamin D, hematopoietic stem cell transplantation, graft-versus-host disease, immune modulation, review

## Abstract

Introduction/Aim: Vitamin D plays a crucial role in immune modulation, which may influence the development of graft-versus-host disease (GvHD) in patients undergoing hematopoietic stem cell transplantation (HSCT). This study aims to evaluate the impact of vitamin D levels and supplementation on the incidence of GvHD in HSCT patients. Methods: A narrative review was conducted across PubMed/Medline, Cochrane Library, CINAHL, and Embase databases. Results: The reviewed studies indicated widespread vitamin D deficiency among HSCT patients, with baseline levels ranging from 12.8 to 29.2 ng/mL. Supplementation protocols varied significantly, with dosages ranging from 1000 IU/day to 60,000 IU/week. Post-supplementation levels improved in some studies. Studies exploring the relationship between vitamin D and GvHD showed mixed results. Lower baseline vitamin D levels were associated with an increased risk of acute GvHD in some studies, while others found no significant correlation. However, a significant association between low levels of vitamin D and the incidence of chronic GvHD was observed. Conclusion: Vitamin D deficiency is prevalent in HSCT patients and may influence the risk of developing chronic GvHD. Future research should focus on larger and more rigorous studies to determine the optimal role of vitamin D as an adjuvant therapy in the context of HSCT.

## 1. Introduction

Hematological diseases encompass a wide range of disorders affecting the blood and its components, such as blood cells, bone marrow, and the lymphatic system [1]. Among these, leukemias, lymphomas, and myelomas are some of the most prevalent and severe conditions [2]. For instance, acute myeloid leukemia (AML) accounts for about 32% of all leukemias in adults, while non-Hodgkin lymphoma has an incidence of approximately 19.1 cases per 100,000 people per year in the United States [3]. Bone marrow transplantation (BMT), also known as hematopoietic stem cell transplantation (HSCT), is a life-saving procedure for many patients with these diseases. This intervention can be autologous, using the patient’s own stem cells, or allogeneic, using stem cells from a compatible donor [4].

Despite significant advances, BMT carries substantial risks and complications, including graft-versus-host disease (GvHD). GvHD is a severe condition where the donor’s immune cells attack the recipient’s tissues, causing damage to organs such as the skin, liver, and intestine [5]. It can manifest in acute or chronic forms and is a major cause of post-transplant morbidity and mortality, with an estimated incidence of 30–70% [6]. Acute GvHD usually occurs within 100 days post-transplant and typically affects the skin, gastrointestinal tract, and liver [7,8]. Chronic GvHD, which can arise months after the transplant, presents symptoms similar to chronic autoimmune diseases and can involve virtually any organ [9].

An emerging issue in patients undergoing BMT is vitamin D deficiency. Vitamin D is essential not only for bone health but also for immune system modulation [10]. HSCT patients are at increased risk of vitamin D deficiency, which can lead to various complications, including bone loss, fractures, and a potentially increased risk of GvHD [11,12]. In an analysis of post-HSCT patients, up to 70% were found to have insufficient vitamin D levels, with an increased risk of osteopenia and osteoporosis, as well as a correlation with higher long-term mortality [13].

Vitamin D plays an immunomodulatory role, influencing various aspects of the immune system, including inhibiting pro-inflammatory lymphocyte subpopulations and promoting anti-inflammatory ones [14]. Vitamin D has pleiotropic effects, acting on different systems of the human body [15]. Apart from its well-known role in regulating calcium metabolism and bone health, vitamin D modulates immune system activity. Preclinical studies have shown that vitamin D inhibits the maturation of dendritic cells (DCs) and favors a Th2 cytokine profile over Th1, reducing the proliferation of allogeneic T cells in response to DC stimulation [16]. This process is associated with increased immune tolerance and a reduced risk of GvHD [17]. Vitamin D also stimulates the production of interleukin-10 (IL-10), an anti-inflammatory cytokine, and reduces levels of interleukin-2 (IL-2) and interferon-gamma (IFN-γ). GvHD is mediated by alloreactive T cells derived from the hematopoietic graft that attack the recipient’s tissues. The presentation of alloantigens by host DCs leads to the activation of donor T cells, which mediate GvHD. Strategies to prevent GvHD include modulating the antigen-presenting capacity of DCs [18,19,20,21]. Vitamin D, due to its immunomodulatory properties, can reduce the risk of GvHD by inhibiting DC maturation and promoting a more tolerant immune environment. In preclinical studies, vitamin D has been shown to inhibit DC maturation, polarize T cell populations towards Th2 cytokine expression rather than Th1, and attenuate allogeneic T cell proliferation in response to DC stimulation [22]. Exposure to vitamin D has led to increased expression of the enzyme IDO (indoleamine 2,3-dioxygenase), responsible for tryptophan metabolism and up-regulated in tolerogenic DCs, suggesting that vitamin D may favor the formation of immature DC populations that promote immune tolerance rather than stimulation.

Current clinical practices for managing vitamin D in HSCT patients are heterogeneous. A survey of allogeneic transplant centers revealed that measuring vitamin D levels is common practice before and after transplantation, with significant variation in the threshold values used to initiate supplementation [23]. Most centers prescribe vitamin D supplements to maintain bone health and enhance post-transplant immune reconstitution. However, there is a discrepancy in monitoring practices and managing vitamin D deficiency, highlighting the need for specific guidelines to harmonize these clinical practices. Notably, 47% of centers regularly measure vitamin D levels before transplantation, and 70% do so after transplantation. Furthermore, 58% of adult centers prescribe vitamin D to maintain calcium metabolism and bone health, while only 24% do so to improve post-transplant immune reconstitution. This indicates an urgent need to standardize clinical practices through evidence-based guidelines. Despite the recognized importance of vitamin D, its role in modulating post-HSCT immunity and preventing GvHD is not yet fully understood [10]. Evidence suggests that adequate vitamin D levels may be associated with a reduced risk of GvHD and improved post-transplant outcomes [24]. However, the evidence is mixed, and further studies are necessary to clarify these effects and determine the best practices for managing vitamin D in HSCT patients.

This review aims to provide a clearer understanding of the role of vitamin D in the context of bone marrow transplantation. Specifically, it seeks to examine vitamin D levels in HSCT recipients during different phases of treatment, identify current vitamin D supplementation regimens, and explore possible correlations between low vitamin D levels and the development and severity of GvHD.

## 2. Materials and Methods

### 2.1. Study Design

A narrative literature review was conducted, building on a previously published methodological study [25], to perform a state-of-the-art review. This type of review aims to summarize the research on a specific topic, highlighting significant changes in understanding and research orientations over time.

### 2.2. Definition of Research Question

The research questions guiding this narrative review were as follows: How do vitamin D levels vary in HSCT recipients during the pre-transplant, immediate post-transplant, and long-term phases? What are the most commonly adopted vitamin D supplementation protocols in patients undergoing HSCT? Is there evidence linking low vitamin D levels with the incidence and severity of GvHD?

These questions were developed using the PICOS framework to ensure a structured and comprehensive approach: P (Population): patients undergoing hematopoietic stem cell transplantation; I (Intervention): vitamin D supplementation; C (Comparison): vitamin D supplementation versus different interventions and/or no interventions; O (Outcomes): identification of optimal vitamin D levels in patients undergoing HSCT and supplementation protocols and determination of any correlation between low vitamin D levels and GvHD; and S (Study Design): primary studies.

### 2.3. Literature Search and Criteria

**Figure 1:**
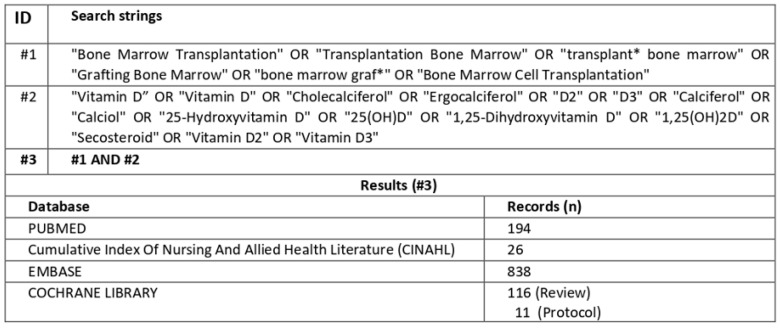
Search strategy.

The search was conducted by consulting the PubMed–Medline, Cumulative Index to Nursing and Allied Health Literature (CINAHL), Embase, and Cochrane Library databases (Figure 1). The inclusion criteria included primary studies published in English conducted on a population of adult patients (18 years and older) affected by hematological disease and undergoing bone marrow transplantation, and studies in which the role of vitamin D related to the onset and/or severity of GvHD was explored. Secondary studies and studies not available in full text were excluded

Following the initial search to identify the total number of records, the article screening process was conducted by two academic researchers (GC and SM). In cases of disagreement between the two, a third researcher (MS) was involved to reach a consensus. EndNote 20 (© 2024 Clarivate, Philadelphia, PA, USA) was used for the bibliographic management of the analyzed records.

### 2.4. Data Extraction and Synthesis

The selected studies underwent a rigorous two-stage analysis process. Initially, they were categorized based on several criteria: author, year, country, type of study, sample, objective, intervention, and results. This categorization ensured a structured approach to synthesizing the identified literature. Following this, a comprehensive narrative synthesis was conducted, integrating the results from different primary study designs. This synthesis offered a holistic perspective on the topic while capturing the nuances and intricacies of each individual study.

## 3. Results

A total of 1159 articles were identified through electronic database searches (PubMed/Medline = 194; CINAHL = 26; Embase = 838; Cochrane Library = 127). Of these, 164 records were duplicates, leaving 1021 to be assessed. After screening titles and abstracts, 955 records were considered irrelevant and 66 articles were screened for full-text. Finally, 57 articles were excluded because they did not meet the defined inclusion criteria, resulting in 9 records being included in this narrative review.

### 3.1. General Characteristics of the Studies Included

This narrative review included three retrospective cohort studies (33.3%) [26,27,28], four observational studies (44.4%) [29,30,31,32], and two retrospective observational studies (22.3%) [33,34] from various countries, published between 2013 and 2023, that examined the impact of vitamin D in patients undergoing HSCT. The included studies originated from the United States [28,29], United Kingdom [30,31], Australia [26], India [33], Turkey [34], Denmark [32], and Sweden [27]. The total number of patients (*n* = 979) ranged from a minimum of 16 to a maximum of 365 (Table 1).

### 3.2. Vitamin D Levels and Supplementation during Different Phases of HSCT

The reviewed studies indicated a widespread deficiency of vitamin D among HSCT patients, either at baseline pre-transplant or during the first evaluation post-hospital admission. The reported baseline levels varied widely, with Bartlett et al. [29] documenting a median of 29.2 ng/mL, while Jindal et al. [33] observed that 86.9% of their sample had levels ≤20 ng/mL. Other studies reported baseline levels ranging from 12.8 ng/mL to 25.6 ng/mL [27,28,32]. One study [34] uniquely included both donors and recipients, reporting baseline levels of 16 ng/mL for donors and 12.8 ng/mL for recipients.

The cholecalciferol supplementation protocols varied among the studies, with different dosages and administration methods. Bartlett et al. [29] used an average dose of 40,000 IU/week, administered via oral thin films (OTF), starting on day 21 until day 428 post-HSCT for over a year. In this study, patients weighing <40 kg received one strip per initial dose, while those weighing ≥40 kg received two strips. The dose was then adjusted based on individual response, with weekly increases or decreases [29]. Another study [33] administered an initial dose of 60,000 IU/week for 8 weeks, followed by a maintenance dose of 800 IU/day orally. Finally, Mastaglio et al. [26] administered 1000 IU/day of cholecalciferol or 0.25 mg of calcitriol per day, starting before HSCT and continuing for one year post-transplant.

Post-supplementation levels showed significant variations. Bartlett et al. [29] reported an increase in vitamin D levels to 58 ng/mL by day 428, while Jindal et al. [33] achieved a notable correction, with 78.3% of patients reaching levels above 20 ng/mL by day 120 post-transplant. Another study [26], although not providing specific post-supplementation levels, supported the need for prolonged supplementation to maintain adequate levels. Other studies [27,28,30,32,34] did not report detailed post-supplementation levels but highlighted the persistent deficiency and its potential impact on HSCT outcomes (Table 2).

### 3.3. Vitamin D Levels and GvHD

Several studies have explored the relationship between baseline vitamin D levels and the risk of developing acute (aGvHD) and chronic graft-versus-host disease (cGvHD) in patients undergoing allogeneic stem cell transplantation (Allo-SCT).

In analyzing aGvHD, two studies [28,31] demonstrated that lower baseline vitamin D levels were associated with an increased risk of developing aGvHD. Specifically, Ros-Soto et al. [31] reported that patients with aGvHD grade 0–II had significantly higher concentrations of 25(OH)D3 compared to those with grade III–IV (41.6 vs. 23.3 ng/mL, *p* = 0.032). Similarly, Glotzbecker et al. [28] observed that patients with baseline vitamin D levels below 25 ng/mL had a higher cumulative incidence of aGvHD grade II–IV at 100 days (53.1% vs. 33.3% in those with levels ≥25 ng/mL, *p* = 0.13). In contrast, two other studies [26,32] found no significant association between pre-transplant vitamin D levels and the occurrence of aGvHD grade II–IV, suggesting that the relationship between vitamin D levels and acute GvHD may not be universal.

Regarding cGvHD, the evidence indicates a significant association with low baseline vitamin D levels. Glotzbecker et al. [28] found that patients with vitamin D levels below 25 ng/mL had a significantly higher cumulative incidence of cGvHD at two years compared to those with levels ≥25 ng/mL (63.8% vs. 23.8%, *p* = 0.009). The hazard ratio for developing cGvHD with low vitamin D levels was 5.26 (*p* = 0.02), indicating a strong association. Similarly, Dikyar et al. [34] demonstrated a negative correlation between baseline recipient vitamin D levels and the development of cGvHD (*p* = 0.011, r = −0.235), further supporting the hypothesis of a link between vitamin D and cGvHD.

Finally, an observational study involving 102 patients [30] indicated a clear link between vitamin D deficiency and the development of GvHD, with approximately 25% of patients developing GvHD. This study also emphasized the need for vitamin D supplementation to mitigate this risk (Table 3).

## 4. Discussion

This study explores the role of vitamin D in the context of HSCT, with a particular focus on evaluating supplementation protocols and the potential correlation between vitamin D levels and the development of GvHD. The primary objective is to determine whether vitamin D may offer a protective effect against GvHD, with the aim of optimizing the clinical management of patients undergoing HSCT. The results [26,27,28,29,30,31,32,33,34] reveal a complex and varied landscape regarding vitamin D management in this patient population. One of the main observations is the widespread vitamin D deficiency, both at baseline before transplantation and in the early post-transplant period. Pre-HSCT levels are generally low, ranging from 12.8 ng/mL to 29.2 ng/mL [27,28,29,32,33,34]. This variability may reflect differences in patient selection criteria, the characteristics of the studied populations, and pre-transplant clinical conditions. Despite this heterogeneity, vitamin D deficiency is a common and significant issue in the management of HSCT patients, aligning with findings in other chronic conditions such as type 2 diabetes and rheumatic diseases, where vitamin D deficiency has been associated with increased inflammation and worsening symptoms [35,36]. For instance, studies have indicated that individuals with darker skin tones, who have higher levels of melanin, are at greater risk for vitamin D deficiency due to reduced synthesis of the vitamin through sunlight exposure [37]. Similarly, older adults often exhibit lower levels of vitamin D, potentially due to decreased skin production and renal function [38]. Furthermore, lifestyle factors such as limited outdoor activity and dietary habits can also contribute to reduced vitamin D levels [39]. These factors should be carefully considered when assessing vitamin D status in HSCT patients, as they may exacerbate the already prevalent deficiency and complicate post-transplant outcomes.

Regarding cholecalciferol supplementation protocols, a significant heterogeneity emerges in both the doses used and the methods of administration. Only two studies [29,33] provide precise indications on the target serum levels of vitamin D to initiate supplementation. Additionally, the doses used vary widely: some studies [29,33] prescribe high weekly doses (40,000–60,000 IU/week), while others [26] opt for more moderate daily doses (1000 IU/day). This lack of standardization in supplementation protocols poses a challenge in the optimal management of this patient population. It is important to note that this deficiency can have significant clinical consequences, such as an increased risk of infections, bone complications, and cardiovascular events [40]. Therefore, careful evaluation of serum vitamin D levels and appropriate supplementation should be an integral part of the routine management of these patients, in order to optimize clinical outcomes and improve their quality of life. A similar situation is observed in other chronic diseases, such as multiple sclerosis and rheumatoid arthritis, where vitamin D deficiency has been associated with an increased risk of developing the disease and greater severity of symptoms [41,42]. In these conditions, as in osteoporosis, recommendations for vitamin D supplementation vary widely due to individual responses and differences in treatment protocols [41,42]. In osteoporosis, in particular, the recommended doses depend on several factors such as age, gender, and the presence of other concomitant medical conditions [43].

The timing of supplementation also varies considerably, with some strategies [33] starting before transplantation and others [29] delaying initiation until the post-transplant period. The importance of prolonged supplementation in maintaining adequate vitamin D levels in the long term has been clearly highlighted [26], but post-supplementation results are variable: some studies [29,33] have reported significant increases in vitamin D levels, while others [27,28,30,32,34] have emphasized the need for continuous supplementation to prevent long-term complications. This variability in approaches suggests the need to develop uniform guidelines.

The results concerning baseline vitamin D levels and the risk of developing GvHD provide important insights for the management of this patient population. Vitamin D plays a crucial role in the immune system, modulating the activity and response of T cells and B cells [14,44]. Therefore, it is reasonable to hypothesize that variations in vitamin D levels may influence the pathogenesis of GvHD, one of the main complications of transplantation. Several studies [26,28,31,32] have investigated the relationship between vitamin D levels and the onset of aGvHD, but the results are not consistent. Some studies [28,31] have found a significant association, suggesting that low vitamin D levels may contribute to a more aggressive immune response and facilitate the development of aGvHD. In contrast, other studies [26,32] have not found such a correlation. This discrepancy may be due to various factors, such as individual patient variability, differences in conditioning and immunosuppression regimens, and specific clinical characteristics of the patients. For example, the impact of vitamin D levels on the pathogenesis of aGvHD may vary depending on the severity of the underlying disease, the presence of comorbidities, or the individual response to immunosuppressive treatment.

Regarding cGvHD, there is evidence showing a strong association with low vitamin D levels. This late form of GvHD, often characterized by autoimmune disorder and multi-organ damage, appears to be influenced by vitamin D, which exerts a protective effect [28,30,34] due to its anti-inflammatory properties and its ability to modulate the adaptive immune response. Specifically, vitamin D can inhibit the differentiation of T cells into pro-inflammatory subtypes, such as Th17 cells, which are often implicated in the pathogenesis of cGvHD. Additionally, vitamin D promotes the function of regulatory T cells (Tregs), which are crucial for maintaining immune tolerance and preventing autoimmunity. This immunoregulatory effect may explain why higher levels of vitamin D are associated with a reduced risk of developing cGvHD [44,45]. Therefore, monitoring and optimizing vitamin D levels may represent a promising strategy for the prevention and management of long-term complications. Vitamin D3 has been shown as having the potential to modulate the immune system by promoting the induction of regulatory T cells via dendritic cell (DC)-mediated pathways [46]. This immunosuppressive effect is particularly relevant given that vitamin D3 enhances antimicrobial defenses by modulating innate immunity and promoting the release of defensins and antimicrobial peptides. These mechanisms could have a beneficial impact on graft-versus-infection (GvI) effects in patients, potentially improving clinical outcomes [46].

Available evidence suggests that while vitamin D may have a significant impact on the risk of developing cGvHD, its influence on aGvHD is less clear and may vary among different patients. The variability in results may stem from differences in study protocols, patient population characteristics, and GvHD diagnostic criteria. Furthermore, vitamin D deficiency may be just one of many factors contributing to the manifestation of GvHD, and its management may require a multifactorial approach.

Available data [34] indicate that vitamin D levels influence not only the HSCT recipient but also have a significant impact on the donor. Observations suggest that vitamin D levels at the time of donation may have a significant impact on transplant outcomes, particularly in the development of GvHD. One study [34] reported that baseline vitamin D levels were on average lower in donors than in recipients, with values of 16 ng/mL for donors and 12.8 ng/mL for recipients. This difference suggests a possible connection between the nutritional status of donors and transplant efficacy, influencing the likelihood of developing GvHD. However, the implications of these relatively low vitamin D levels in donors remain unclear. Further investigation into vitamin D levels in donors could provide insights into how to improve pre-transplant management. If donors with vitamin D deficiencies have a higher risk of compromising transplant outcomes or contributing to a more aggressive immune response, it may be beneficial to consider strategies for supplementation or optimization of vitamin D levels in donors as well.

Looking to the future of cancer and the management of associated complications, the role of vitamin D represents a promising area of research with potential significant clinical implications. Current data suggest that vitamin D could be used as an adjuvant therapy to mitigate the risk and severity of GvHD, particularly in its chronic form (cGvHD). Its immunomodulatory and anti-inflammatory properties provide a solid basis for considering vitamin D supplementation as an integral part of therapeutic strategies aimed at improving post-transplant outcomes and patients’ quality of life.

### Limitations and Future Perspectives

This review presents several limitations. The limited availability of high-quality randomized controlled trials (RCTs), small sample sizes, and the variability in vitamin D supplementation protocols in the included studies introduce significant challenges. These limitations prevent drawing definitive conclusions and may hinder the development of standardized protocols for vitamin D supplementation in patients undergoing HSCT. Future research should prioritize larger and more methodologically rigorous studies that standardize the dosage and timing of vitamin D administration according to international guidelines. It is also essential to consider and control for potential sample-related biases, such as associated comorbidities, lifestyle factors, and racial variables.

Furthermore, it is crucial to examine the potential effects of vitamin D on the nutritional status of donors, given its influence on transplant outcomes. By addressing these gaps, vitamin D supplementation could be validated as a key component of adjuvant therapies, potentially reducing transplant-related complications, minimizing the need for aggressive immunosuppressive treatments, and improving long-term patient survival.

## 5. Conclusions

This study highlights the potentially significant role of vitamin D in the management of patients undergoing HSCT, with particular attention to the prevention of GvHD. The evidence gathered indicates a widespread deficiency of vitamin D in this population, both before transplantation and in the post-transplant period. Despite the variability in supplementation protocols and the lack of standardization in dosages and timing, the need for careful monitoring of vitamin D levels and adequate supplementation to improve clinical outcomes emerges. The associations between vitamin D levels and the development of GvHD are complex and vary between the acute and chronic forms of the disease. While there is a widespread deficiency of vitamin D in this population, the link with acute and chronic GvHD appears less defined, suggesting the need for further research to clarify these relationships. Moreover, the potential impact of vitamin D levels in bone marrow donors represents an emerging area of study that could influence future research and pre-transplant management strategies.

In light of these observations, future research should prioritize larger and more rigorous studies to determine the optimal role of vitamin D as an adjuvant therapy in the context of HSCT. If its efficacy is confirmed, vitamin D supplementation could become a crucial component in preventing post-transplant complications, improving patients’ quality of life, and reducing the need for more aggressive immunosuppressive treatments.

## Figures and Tables

**Table 1:** Characteristics of the studies included.

Author, Year, Country	Type of Study	Sample	Objective	Intervention	Results
Bartlett et al. 2023 USA [9]	Observational study	20 (Allo-SCT)	Increase vitamin D levels	Cholecalciferol supplementation	Improvement in vitamin D levels; no toxicity observed
Ros-Soto et al. 2022 UK [31]	Observational study	16 (Allo-SCT)	Vitamin D levels and GvHD	Measurement of VitD and GvHD biomarkers	Association between patients vitamin D levels and GvHD
Jindal et al. 2022 India [33]	Retrospective Observational study	162 (Allo-SCT)	Vitamin D levels and GvHD	Cholecalciferol supplementation	High incidence of vitamin D deficiency; no association between GvHD and vitamin D levels
Gjærde et al. 2021 Denmark [32]	Observational study	116 (Allo-SCT)	Vitamin D levels and GvHD	Measurement of VitD levels	No significant association between vitamin D levels and acute GvHD
Dikyar et al. 2020 Turkey [34]	Retrospective observational study	123 donor; 123 recipents (Allo-SCT)	Vitamin D levels and GvHD	Evaluate the possible impact of donor and recipient VitD levels on HSCT outcome	Association between patients vitamin D levels and GvHD
Mastaglio et al. 2019 Australia [26]	Retrospective matched cohort study	IG 78 CG 156 (Allo-SCT)	Vitamin D levels and GvHD	Cholecalciferol supplementation	No association between patients vitamin D levels and GvHD
Quillinan & Murray 2019 UK [30]	Observational study	102 (Allo-SCT)	Vitamin D levels and GvHD	Measurement of VitD levels	Clear link between VitD deficiency and GvHD; 25% of patients developed GvHD; need for VitD supplementation
Von Bahr et al. 2015 Sweden [27]	Retrospective cohort study	166 (Allo-SCT)	Vitamin D levels and GvHD	Measurement of VitD levels	Low baseline vitamin D levels
Glotzbecker et al. 2013 USA [28]	Retrospective cohort study	53 (Allo-SCT)	Vitamin D levels and GvHD	Measurement of VitD levels	Low vitamin D levels associated with increased risk of chronic GVHD

Legend: Allo-SCT = allogeneic stem cell transplantation; GvHD = graft-versus-host disease; VitD = vitamin D; HSCT = hematopoietic stem cell transplantation; IG = intervention group; CG = control group.

**Table 2:** Vitamin D levels and cholecalciferol administration.

Author	Sample (*n*)	Cholecalciferol Start Indication	Cholecalciferol Dosage (Mean)	Method of Administration	Time of Administration	Vitamin D Levels(ng/mL) [Timing]
Bartlett et al. [9]	Allo-SCT (*n* = 20)	≤35 ng/mL	40,000 IU/week	OTF	From day 21–428 post-transplant	t0: 29.2 [+21] t1: 53 [+51] * t2: 58 [+428]
Jindal et al. [33]	Allo-SCT (*n* = 162)	≤20 ng/mL	60,000 IU/week	Oral	For 8 weeks followed by maintenance with 800 IU/day	t0: ≤20 [pre-HSCT] (86.9%) t1: N.R t2: 34 [+120]
Mastaglio et al. [26]	Allo-SCT (*n* = 78)	N.R	1000 IU/day or 0.25 mg calcitriol/day	Oral	Before HSCT until 1 year post-transplant	N.R
Gjærde et al. [32]	Allo-SCT (*n* = 116)	N.R	N.R	N.R	N.R	t0: 25.6
Dikyar et al. [34]	Donors ^a^ (*n* = 123); Allo-SCT ^b^ (*n* = 123)	N.R	N.R	N.R	N.R	t0: 16 ^a^ 12.8 ^b^
Quillinan & Murray [30]	Allo-SCT (*n* = 102)	N.R	N.R	N.R	N.R	t0: ≤20 [pre-HSCT] (73.5%)
Von Bahr et al. [27]	Allo-SCT (*n* = 166)					t0: 15.6 [pre-HSCT]
Glotzbecker et al. [28]	Allo-SCT (*n* = 116)	N.R	N.R	N.R	N.R	t0: 21.9

Legend: t0 = hospital admission/pre-transplant; t1 = day 30; t2 = variable timing; OTD = oral thin film; Allo-SCT = allogeneic stem cell transplantation; HSCT = hematopoietic stem cell transplantation; N.R = not reported; * after 4 weeks of baseline t0; ^a^ Donors; ^b^ Allo-SCT.

**Table 3:** Baseline vitamin D levels and GvHD.

Author, Year	Sample	aGvHD Results	cGvHD Results
Ros-Soto et al. [31]	Allo-SCT (*n* = 16)	Patients with grade 0–II had a higher concentration of 25(OH)D3 compared to those with grade III–IV (41.6 vs. 23.3 nmol/L; *p* = 0.032)	N.R
Mastaglio et al. [26]	Allo-SCT (*n* = 78)	No association between baseline vitamin D levels and GVHD	
Gjærde et al. [32]	Allo-SCT (*n* = 116)	No association between baseline vitamin D levels or vitamin D insufficiency and acute GvHD	N.R
Dikyar et al. [34]	Donors (*n* = 123) Allo-SCT Recipients (*n* = 123)	N.R	Negative correlation between baseline recipient VitD levels and cGvHD (*p* = 0.011, r = −0.235)
Quillinan & Murray [30]	Allo-SCT (*n* = 102)	Association between GvHD and baseline vitamin D deficiency with approximately 25% of patients developing acute or chronic GvHD	
Glotzbecker et al. [28]	Allo-SCT (*n* = 116)	aGvHD grades II–IV at 100 days was 53.1% in patients with vitamin D < 25, versus 33.3% in patients with vitamin D ≥ 25 ng/mL (*p* = 0.13)	Low baseline vitamin D levels are associated with cGVHD (hazard ratio = 5.26, *p* = 0.02)

Legend: aGvHD = acute graft-versus-host disease; cGvHD = chronic graft-versus-host disease Allo-SCT = allogeneic stem cell transplantation; baseline = pre-transplant or hospital admission; 25(OH)D3 = 25-Hydroxyvitamin D3; N.R = not reported.
